# Impact of Seasonality on Physical Activity: A Systematic Review

**DOI:** 10.3390/ijerph19010002

**Published:** 2021-12-21

**Authors:** Antonio Garriga, Nuria Sempere-Rubio, María José Molina-Prados, Raquel Faubel

**Affiliations:** 1 Faculty of Physiotherapy, Universitat de València, 46010 Valencia, Spain; angarhifis@gmail.com; 2 Department of Physiotherapy, Universitat de València, 46010 Valencia, Spain; maria.jose.molina-prados@uv.es (M.J.M.-P.); raquel.faubel@uv.es (R.F.); 3 Clinical Biomechanics Research Unit (UBIC), Department of Physiotherapy, Universitat de València, 46010 Valencia, Spain; 4 Joint Research Unit in IctAapplied to Reengineering Socio-Sanitary Process, IIS La Fe—Universitat Politècnica de València, 46026 Valencia, Spain; 5 PTinMOTION—Physiotherapy in Motion Multispeciality Research Group, Department of Physiotherapy, Universitat de València, 46010 Valencia, Spain

**Keywords:** physical activity, health promotion, seasonality, sedentarism

## Abstract

Background: The purpose of this study was to collect and analyze the available scientific evidence of the impact of seasonality on physical activity (PA). PA refers to walking, biking, sports and/or active recreation. Methods: The search was performed in the following databases: PubMed, PEDro, Cochrane and Embase. All publications from January 2015 to September 2020 assessing seasonal variations on physical activity development in adults were selected. Results: A total of 1159 articles were identified, of which 26 fulfilled the selection criteria involving 9300 participants from 18 different countries. The results obtained suggest that seasonality affects PA independently of the countries, pathologies of the participants and the tool to collect PA information. Conclusions: PA level varies across the seasons, with higher PA level in summer compared with other seasons, especially in winter. Sedentary behavior follows the opposite trend. Impact of seasonality variations should be considered in clinical research involving PA as a primary outcome as well as in interventions on PA promotion.

## 1. Introduction

The World Health Organization (WHO) defines physical activity (PA) as “any bodily movement produced by skeletal muscles that require an expenditure of energy. Physical activity refers to all movements, including during leisure time, for transportation to and from places, or as part of a person’s work. It considers sports that can be practiced at any level as: walking, biking, active recreation, and different games” [1]. 

The WHO recommends 150 min of moderate-intensity physical activity (PA) per week in adulthood and old age [2]. However, the percentage of the world’s population that does not reach the minimum levels of PA is still high [3]. Precisely, according to the WHO, 23.3% of the global population in 2010, and 27.5% in 2016 [3,4]. One out of four adults is not active enough. In terms of the geographical zone, the US (32%) and Eastern Mediterranean regions (31%) [5] exceed the world average. As well, in the European Union (EU)—two thirds of the population does not reach the minimum recommendations for adults [6,7]—and the Arabic region the inactivity rates are over 60% [8].

Physical inactivity is a global problem [9,10,11] that generates a growing concern [5]. It contributes to the obesity epidemic [12,13], and increases morbidity and mortality rates in chronic diseases [5,14,15,16,17,18]. It is associated with disease exacerbations, increased pain, poorer health-related quality of life and prognosis, among others health outcomes [15,19,20,21,22,23,24,25,26].

For this reason, public health experts have turned their attention to the promotion of PA for its multiple health benefits [14,27]. Recently, the number and relevance of PA promotion interventions have increased such as PA promotion strategy for the WHO European Region 2016–2025 [28]. It is now well-know that there are factors that modulate participation in PA promotion programs, such as place of residence [29], accessibility to facilities [30,31], socio-economic characteristics of individuals [32], lifestyle [33], aging population [34], previous pathologies [19,35], beliefs and values [36,37], etc. [38]. One of this PA determinant could be seasonality [34], defined as the natural periods that the year (spring, summer, autumn and winter) [39].

Seasonality seems to impact PA levels and the exacerbations of some diseases and mortality [29,34]. Temperate climates have revealed higher mortality rates in winter than summer [40,41]. Seasonality is also known to affect more the aging people [29,34]. Moreover, in populations with pathologies, the risk of exacerbations increases during winter [15]. For example, preoperative lung cancer patients are much less physically active in the winter season, affecting their functional capacity. Thus, they could not be considered suitable for some surgical interventions during winter months [42]. In addition, adherence decreases in diseases such as COPD and HF during summer months. [15,43]. Additionally, due to the lack of adapted indoor facilities, wheelchair users are affected by seasonal variations and unfavorable weather conditions [44].

In recent years, PA promotion programs [2,3,27] have increased worldwide. However, despite the considerable heterogeneity of environmental conditions, there is little research on their influence on PA. Moreover, current guidelines and consensus do not adapt to the different periods of the year and the challenges they pose for PA implementation [45]. In recent years, the concern about climate change and global warnings has increased, including the analysis of its impact on health [46]. Accordingly, there has been increasing attention toward consideration of such change as a barrier to physical activity, including interest in other variables that could be modified by it, such as seasonal variations [47]. 

As humans cannot modify meteorological and seasonal conditions at their own will, the most intelligent response is to understand better how they affect PA to adapt and reduce or stop the adverse impact on the PA levels populations [42]. Being aware of how weather conditions affect physical activity can help policymakers and healthcare providers to adopt recommendations to mitigate its effects [47]. Thus, collecting data on PA and seasonality is crucial because it provides information on what strategies and interventions need to be modified during the different seasons of the year to avoid physical inactivity [39]. 

The aim of this systematic review is to compile and evaluate current available evidence about the impact of seasonality on PA and to describe the different strategies and tools used to collect variables related to PA.

## 2. Materials and Methods

This study follows the guidelines of the Preferred Reporting Items for Systematic reviews and Meta-Analysis (PRISMA) [48]. The Supplementary Table S1 provide the details of domain-specific score.

### 2.1. Research Strategies

The systematic search was executed using a structured electronic search in PubMed, PEDro, Cochrane and Embase databases in the October–December 2020 period. PA and seasonality have been the main two elements of search. Both on MeSH Terms (motor activity, exercise, training and seasons) and free terms keywords (physical activity, season), (Supplementary Table S2). Truncation has also been used for the keyword “season”. A manual search which included the references and related articles, has been also carried out.

### 2.2. Selection Criteria

Articles published in any language between January 2015 and September 2020 (both included) assessing the influence of seasonality on PA were included. Concerning exclusion criteria, systematic reviews, meta-analysis, case studies and those studies conducted with participants under the age of 18 were excluded. Similarly, studies evaluating the influence of weather instead of seasonality were also excluded. Lastly, we excluded studies that do not differentiate between established standard seasons (e.g., referring to rainy/non-rainy seasons, school year vs. summer holidays) or those that measuring PA only within a season.

### 2.3. Assessment of Methodological Quality

For the quality assessment, the “standardized instruments from the Joanna Briggs Institute System for the Unified Management, Assessment and Review of Information” (JBI SUMARI) checklist was used to report and critically appraise the methodological aspects of included studies [49]. These instruments included the JBI Critical Appraisal Checklist for Comparable Cohort, the JBI Critical Appraisal Checklist for Cross-sectional Studies, the JBI Critical Appraisal Checklist for Randomized Control Trial [50] and the JBI Critical Appraisal Checklist for Quasi-Experimental Studies, and were chosen accordingly to the study design [49,50,51].

### 2.4. Data Extraction

Climate information was collected according to Köppen climate classification, first published in 1936. This instrument classifies climate into five main classes: tropical zone (A), arid zone (B), temperate zone (C), snow zone (D), and polar zone (E). The five main climate classes are further subdivided into 30 climate subtypes. Each subtype is defined by two or three letters code: the first letter is referred to the main class of climate, the second letter indicates the seasonal precipitation type, while the third letter indicates the level of heat [52].

The variables included in Table 1 and Table 2 were gathered, such as: country of implementation, climate related information, objective of the study and year of publication, characteristics of the participants (age, sex, chronic diseases, and other relevant information about the population), seasons, tools for collecting information on PA, measurement time of each outcome and PA results for each included study. Two different reviewers selected studies, rated methodological quality, and extracted data independently. If there were any disagreements between both investigators, a third independent researcher determined inclusion/exclusion.

## 3. Results

### 3.1. Search Results

**Figure 1:**
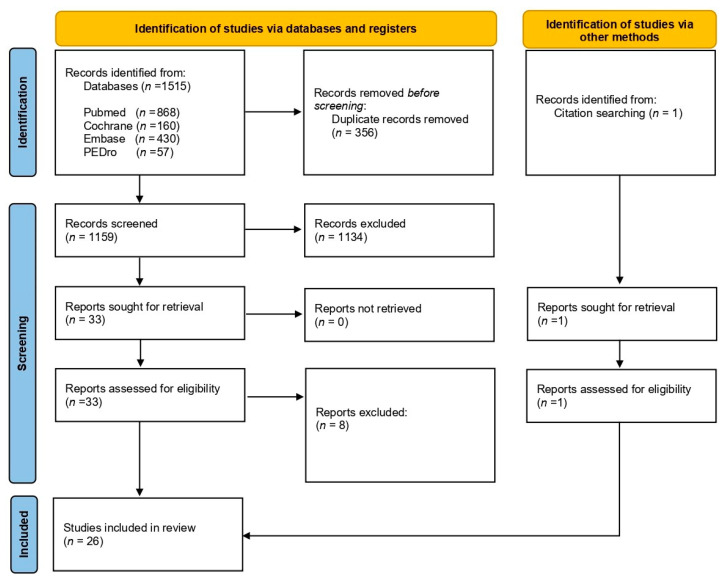
Flowchart of study selection process.

As shown in the PRISMA flow diagram (Figure 1), after the initial search and eliminating duplicates, 1159 articles were identified, of which 1126 studies were eliminated after reading the title and summary. Of the 33 remaining, after critical reading of the complete text and including one reference from manual search, 26 studies were finally selected for the systematic review.

Concerning the quality appraisal, cohorts studies were over 63% of the items achieved, except for one study [55]. Items related to missing data and lost follow-up were not achieved in any of the studies. Regarding cross-sectional studies, all studies were over 75% and three of them accomplished 100% of the criteria. Lastly, RCT were over 60% of the items achieved. None of them blinded participants to treatment assignment. The details of domain-specific score are provided in Supplementary Tables S3–S5.

### 3.2. Characteristics of the Included Studies

The characteristics of the studies included are detailed in Table 1 and Table 2. These 26 studies were conducted in 18 different countries with a dominance of the USA with six contributions [16,20,23,24,27,53] and Canada with four [9,31,54,56]. Two studies were developed in countries as Japan [13,55] and Poland [17,44]. Other countries developed one as: France [59], Iceland [37], Lithuania [32], Netherlands [34], Qatar [5], South Korea [42] Sweden [45], and the United Kingdom [57]. Three studies were carried out with participants from 2 or more countries: Belgium and Brazil [19], Norway, Denmark and Australia [15], and the Netherlands and Switzerland [60].

Regarding types of climates, according to the Köppen climate classification, 9 types of climates were represented in the selected articles. Most of the studies were developed in locations with Dfb climate (11 studies) corresponding to a warm-summer humid continental climate [23,24,31,32,34,44,45,53,54,56], 5 studies in Cfb regions (temperate oceanic climate) [15,19,57,59,60], 4 in Dfc (subartic climate) [15,45,54,56] and 3 in Cfa (humid subtropical climate) [13,19,55]. Just one study corresponds to a Bwh (hot desert climate) [5], Cfc (subpolar oceanic climate) [37], Dfa (hot summer humid continental climate) [20] or Dwa (Monsoon-influenced hot-summer humid continental climate) [42].

A total of 9300 people took part in the set of studies collected by this review. There was a great variability between the sample sizes. Four studies included more than 1000 participants and nine less than 100 participants. Regarding the age of the people included, the mean age ranged from 30 years [44] to 80.3 years [37]. Twelve out of the 26 publications included participants with a specific pathology: chronic obstructive pulmonary disease (COPD) [15,19,20,59,60], heart failure (HF) [23,24,45], lung cancer [42], coronary heart disease [56], spinal cord injury [44] and type II diabetes and/or hypertension [54].

Concerning the design of the included studies, 15 were observational longitudinal studies (cohorts), including prospective (14 studies) [5,9,19,27,37,42,44,53,54,55,56,57,59,60] and one retrospective design [24]. Seven were designed as cross-sectional observational studies [13,15,17,31,32,34,45], one study was a quasi-experimental pre-post design [58], and three out of the 26 studies were randomized controlled trials [16,20,23]. On the other hand, regarding the objective of the study, most of the studies were specifically developed to examine the impact of seasonality on PA; only six out of the 26 articles considered seasonal variations as a secondary part of the research.

### 3.3. Physical Activity Collection Instruments

The articles included used different tools (objective or subjective) for PA information collection. Twenty-one out of the 26 studies employed objective (direct) methods. Meanwhile eight used subjective methods through questionnaires or self-reported variables, either exclusively [31,32,44,45,60] or together with objective methods [13,17,54]. Among the objective tools, there is a preference for pedometers [5,9,16,17,20,27,55,56], and accelerometer, [13,23,24,34,37,54,57,58]. Other instruments like activity trackers [15,19,42], smartwatch [53] or actimeters [59] were also employed. The subjective tools were different questionnaires like IPAQ either the short [45,54] and the long form [17], GPAQ [32], PASE [13], LTPAQ [44], LASA-PAQ [60] or specific questionnaires for active transportation [31,32].

In this line, there were different outcomes variables to describe PA level: the most frequent was the total number of steps, employed in 16 studies. In seven studies, it was the time spent in physical activity according to the intensity of PA [5,13,15,16,17,19,23,34,37,42,58]. Others included different related variables such as total energy expenditure (TEE) [13,15] or active transportation [31,32]. Finally, five studies also measured sedentary behavior [15,34,37,54,58].

Regarding measurement times, 13 out of the 21 studies that worked with objective methods applied 1 week as the period of physical activity data collection [9,13,15,17,19,20,34,37,42,54,55,56,59]. Eight studies [5,13,16,23,24,27,53] compiled data continuously during the length of the follow-up. Additionally, there were subjective methods, as questionnaires, passed once per season.

### 3.4. Physical Activity and Seasonality

**Figure 2:**
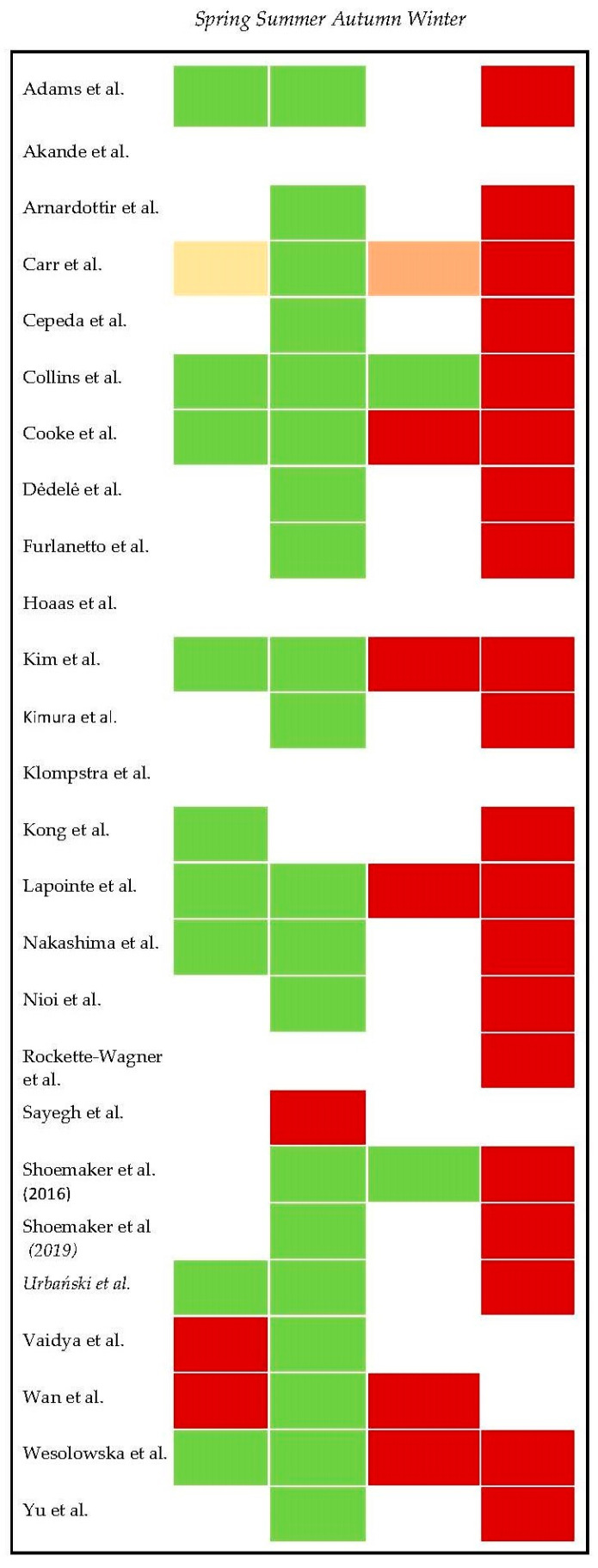
Statistically significant variations of physical activity (PA) according to seasons (green color for highest PA and red for lowest PA in each study).

Most of the studies (22 out of 26) found significant variations in PA among different seasons. In contrast, three [9,15,45], noticed no significant differences in PA related to seasonality. The three that found no significant differences were included in countries with low winter temperatures (Canada, Norway, Denmark, and Sweden) with average minimum temperatures in winter from −15 to −37 °C. However, the study conducted by Sayegh et al. [5], in a subtropical desert climate with very high summer temperatures and humidity, showed a significant decrease in PA in summer. Figure 2 clarifies graphically the results regarding statistically significant variations of physical activity (PA) according to the seasons.

Overall, studies showed a higher level of PA in summer compared to winter and compared to all other seasons. Some studies compared seasons more favorable for PA, spring/summer vs. autumn/winter, finding statistically significant differences. Others works discovered that spring is the season of the year with the highest level of physical activity. Finally, a few studies noticed significant decreases in PA in winter compared to the rest of the seasons.

Some of the included studies showed results disaggregated by age group. Cepeda et al. [34] observed that middle-aged participants (50–64 years) and young elderly (65–74 years) were more physically active in summer than in winter. Meanwhile, the elderly (≥75 years) displayed no seasonal variations. Finally, Wesolowska et al. [17] remarked a higher number of steps in summer and spring compared to winter in all age groups.

Regarding the level of activity of the participants, Arnardottir et al. [37] noticed more physical activity in summer and, according to the stratified results, this summer-winter difference was significantly more elevated in the high activity level group than the low activity group. In the same line, Shoemaker et al. [24] also found a greater impact on seasonality in participants with fewer comorbidities and with physical activity longer than 2.2 h per day. Lapointe et al. [56] discovered that seasonal variations influence physical activity, but only in the active group (lower physical activity in autumn and winter than in spring and summer). For the low-activity group, no significant differences between seasons were observed. Nevertheless, in the study by Kim et al. [27] carried out on women, members of the active group were more likely to maintain the increase in the number of steps achieved in spring at the arrival of autumn–winter, contrary to women in the other two groups (low active and somewhat active), that showed a significant decrease. 

Concerning sedentary behavior (SB): only three out of five studies assessing SB, found statistically significant differences: more sedentary time in winter compared to summer [37,58] and in autumn–winter compared to spring–summer [54].

## 4. Discussion

### 4.1. Seasonal Variations on Physical Activity

This systematic review identified 26 of 1159 articles that fulfilled the selection criteria to determine the impact of seasonality on PA. Overall, the results of the review showed that PA increases significantly in the summer-spring months compared to winter. It occurs independently of the countries’ climate, the characteristics and previous pathologies of the participants. Regarding the geographical areas, results were similar despite the climate of the region except for the sub-desertic climate where PA level decreased in summer.

Seasonality variations on PA appeared in the included studies in this systematic review independently of whether they are performed in healthy participants or in subjects with specific pathologies such as COPD, heart failure, lung cancer, coronary heart disease, spinal cord injury, type II diabetes and/or hypertension.

According to variations within the states, some countries like the United States, Canada and Australia cover immense land areas. It means that they certainly have a wide range of climates and seasonal variations. On the contrary, Scotland, Netherlands and France do not have as much land extension; however, there are discrepancies due to urban-suburban, mountain-coast or north-south disparities such as in the Scandinavian countries where there are many distinctions concerning the daily sunshine hours in the same territory [39]. Consequently, these countries may also require specific studies in different areas, as the geographical location is as important as the size of the nation.

Exploring the three selected studies that do not find statistically significant differences, two out of the three do it with clinically significant differences in practice. For instance, the study developed by Hoaas et al. [15] on COPD patients from Norway, Denmark and Australia, shows an increased number of steps at all locations during summer compared to all other seasons. This increased number of footsteps, furthermore, exceeds the minimum clinically important difference established for COPD patients (between 600 and 1100 footsteps/day). The magnitude of this PA growth is related to a reduced risk of hospital admissions [61]. Nevertheless, this difference is not statistically significant probably due to modest sample size in each group. In addition, the study conducted by Klompstra et al. [45] in participants with heart failure from Sweden, stablishes a change of 250 METs/week as clinically significant and some of the patients increased their PA in summer over this amount. Unexpectedly, this study observes that a large percentage of participants increased their PA in winter compared to summer. Authors concluded that his variation may be explained because the differences between summer and winter temperatures during the study were not as marked as expected and also the low response of the participants in the winter period (58%) may have caused a response bias. On the contrary, Kim et al. found statistically significant -but small in magnitude- differences in PA among seasons with a foreseeable little clinical impact as they do not exceed 300 steps/day.

According to the results of our review, the level of physical activity of the participants may be relevant, in seasonal variations of PA showing a greater impact of seasonality in participants with a higher level of PA [24,27,37,56]. In those population, the increase of PA activity in spring-summer is bigger than in low PA level participants. In this point of view, the results imply that participants who are physically inactive may not change their level of PA even in more favorable season.

Regarding different levels of intensity of PA, some studies collected them separately and their results could be relevant. For example, Cepeda et al. [32] found that the greatest seasonal variation was in light PA levels. Likewise, in the work of Furlanetto et al. [18] in patients with COPD, they found no differences in activities from moderate to vigorous intensity (above 3 METs). Nevertheless, they found differences in the time spent in activities with an intensity above 2 METs and suggest that this could be a more appropriate measure for subjects with a low activity profile.

Results of this work are consistent with a previous systematic review published in 2007 including a total of 37 studies between 1980 and 2006 [37]. The findings showed the level of PA also varies with seasonality, being higher in spring and summer and lower in winter. 27 out of the 37 studies included in the review found statistically significant differences in PA. Nevertheless, this previous review only included studies developed in 8 different countries showing a less range of diversity of territories. In addition, it analyzed together the impact of both, weather and seasonality, on PA. Additionally, a more detailed analysis is required in order to take into account stratification about age groups or level of PA.

### 4.2. Tools for Physical Activity Assessment

Studies included in the systematic review use different tools for PA information collection. In general, the results remain the same despite the tools used to collect PA both direct (objectives) and subjective instruments. However, some publications that applied both objective and subjective methods assessed if there was a correlation between different methodologies. Both Cooke et al. [54] and Wesolowska et al. [17] found that there was no correlation between objective and subjective methods. In fact, Wesolowska et al., in the absence of a statistically significant correlation between pedometer values (objective) and IPAQ scores (subjective), suggested that the participants may not correctly assess their own level of physical activity using subjective tools [17].

The use of objective methods seems to display numerous advantages as they measure the changes in physical activity and sedentary behavior more accurately than questionnaires [62,63,64]. They offer objective feedback [17,34], encouraging the interruption of long periods of sedentary behavior. Even in real time, they facilitate compliance with physical activity goals. They contribute as well, to design interventions in physical activity promotion and sedentary behavior [34]. They bring to the patients the opportunity to work out when they have contraindications to strenuous exercise. Additionally, they help positively in those who are less motivated to move and procure reproducible results [17].

Nevertheless, some objective methods (i.e., accelerometer) are not very accurate in distinguishing activities that fluctuate according to seasonal patterns such as cycling or swimming, with higher practice in summer. Thus, studies that rely purely on the use of accelerometers may then underestimate seasonal differences [65]. Objectives tools are also restricted estimating upper body movements during transport and heavy lifting activities [37]. More importantly, it may bias the assessment of the level of PA due to its effect on the promotion of physical activity. Nevertheless, most of the studies that incorporated objective methods used 1-week measurement periods which give a reproducible and practical dimension of physical activity and sedentary time [66]. Limiting the use of pedometers or accelerometers to specific weeks would help to reduce their effect/impact on the results. This strategy may be beneficial for some research, but also counterproductive or limiting, as it reduces the ability to identify variations in PA throughout the year due to the influence of external factors such as seasonality [39,67,68].

Subjective methods are commonly used to collect PA information because of their low cost, easier and faster administration, compared to direct methods and their possibility to measure different types of activities [32]. Furthermore, compared to other methods such as pedometers or accelerometers, they have a low level of influence on the results [67]. However, regarding the limitations of subjective methods, they might lead to an erroneous estimation of the activity performed [17,32] and may not detect seasonal variation while objective methods have done so, as in the study by Cooke et al. [54].

### 4.3. Study Designs for Seasonality Assessment

Due to the one-year periodic variations, the optimal way to analyze seasonality would be through a longitudinal study using the same individuals—if possible, for more than 1 year, to reveal parallel and divergent trends between years with more or less adverse climatic conditions [44]. Drawing conclusions about seasonality based on non-longitudinal designs, which compare different groups of people from different seasons with short sampling periods, may not be the most appropriate design [24].

Cross-sectional collection methods do not provide information on trajectories at the individual level [16], neither, do they observe the changes in PA of the same group of patients in different seasons. If in addition to being a cross-sectional model, there is a deficit in the number of patients recruited in one or more seasons, and the results regarding seasonal variations in physical activity could be influenced. As in the work performed by Hoaas et al. [15], where the low number of physical activity data collected during summer could have influenced the results; or in the study by Kong et al. [42] and the study conducted by Klompstra et al. [45], where the number of patients observed in winter is limited.

### 4.4. Limitations and Strengths of the Review

The present review was conducted following the PRISMA checklist. One of the limitations was the heterogeneity of the tools for the PA assessment, sample size, characteristics of the population and measurements periods. At the same time, the consistency of the results even with this heterogeneity represents the magnitude of the impact of seasonality on PA levels. The studies have been carried out in 18 different countries in the two hemispheres and on four continents.

Additionally, there are other constraints: (a) the small sample sizes of some of the studies [13,19,23,55,56,57]; (b) the low number of articles carried out in two or more countries and studies showing results disaggregated by age and gender population groups; (c) some of the included studies are conference abstracts [53,54,56,58,59,60], although there was some sufficient information for the synthesis of results; (d) limited date range for study publication; (e) regarding the study design, most of the publications are observational studies both cohorts (15 studies) or cross-sectional (seven studies) with only four studies with a quasi-experimental [58] or experimental design [16,20,23]. Given the characteristics of the research question, it was expected that most of the designs were observational. 

Future studies on the effects of seasonality on PA are, in general, required with the widest possible diversity of locations, pathologies and population groups and with the application of the most appropriate methodologies to capture and quantify seasonal variation.

### 4.5. Implications of the Results for Clinical Research and PA Promotion Interventions

As the results of this systematic review showed that the influence of seasonal variations on physical activity is relevant for the general population—and certain groups—it should always be considered as one factor that may influence PA outcomes. In both, PA promotion interventions and clinical research, seasonality should be perceived as one of barriers for users to join in physical activities.

The results of this review may be helpful to identify the better time to set up or change the physical activities for people. It may also be useful, to implement PA maintenance strategies in seasons with a tendency to reduction (autumn–winter). This includes initiatives on the environment and facilities that allow opportunities to perform PA despite the characteristics of the different seasons. On the other hand, establishing light PA strategies might replace the increased sedentary time during winter and autumn, which suffers the greatest seasonal variation [34]. It can also be productive to incorporate specific and individualized education according to the season. Moreover, depending on the environmental context, encourage PA regardless of seasonal changes.

The results may also be useful for the interpretation of PA assessments. This is particularly relevant, where there may be differences in seasonal variations either because the research has been carried out in several countries or in a single one with significant variations across the country. In research studies it will be essential to take into account the effect of seasonality in both the initial and subsequent measurements during the follow-up period. It will be important to be careful when extrapolating PA results to locations with different seasonality conditions.

## 5. Conclusions

PA level follows seasonality variations finding higher PA level on summer compared with other seasons, especially on winter. Sedentary behavior follows the opposed trend as PA level regarding seasonality. Results are consistent in different countries and populations with chronic diseases, but future studies are required to get more detailed about its impact on gender and different age ranges or previous intensity of PA level of the individuals. Impact of seasonality variations should be considered in clinical research involving PA as a primary outcome and necessarily for interventions on PA promotion. Public health interventions could be implemented in order to analyze the potential impact of seasonality as a barrier for PA development in each specific context.

## Figures and Tables

**Table 1:** Descriptive characteristics and results of the included studies.

Author (Year)	Country	Climate Data	Objective	Study Design
Adams et al. (2018) [53]	USA (Vermont)	Dfb: Warm summer humid continental	To examine seasonality impact on PA	Observational longitudinal prospective study (cohort)
		Average T (°C):		
		Summer (max): 26		
		Winter (min): −14		
Akande et al. (2019) [9]	Canada (Nunavut)	ET: Tundra	To measure physical activity levels and explore factors influencing PA	Observational longitudinal prospective study (cohort)
		Average T (°C):		
		Summer (max): 7		
		Winter (min): −37		
Arnardottir et al. (2017) [37]	Iceland	ET/Cfc: Tundra/subpolar oceanic	To examine seasonality impact (hours of daylight) on PA	Observational longitudinal prospective study (cohort)
		Average T (°C):		
		Summer (max): 12		
		Winter (min): −7		
		Natural light (h) = 14/7		
Carr et al. (2016) [16]	USA	-	To explore the variability of physical activity	Randomized controlled trial
Cepeda et al. (2018) [34]	Netherlands (Rotterdam)	Dfb: Warm summer humid continental	To examine the seasonality of daily levels of PA	Cross-sectional study
		Average T (°C):		
		Summer (max): 22		
		Winter (min): 0		
		Heavy rain during spring and autumn		
Collins et al. (2015) [31]	Canada (Ontario)	Dfb: Warm summer humid continental;	To assess the prevalence, mode, destinations, and duration of active transportation	Cross-sectional study
		Max. Temp (°C): −2.8 in winter, 10.9 in spring, 25.7 summer, and 13.3 in autumn		
Cooke et al. (2016) [54]	Canada (Montreal)	Dfb/Dfc: Warm summer humid continental/subarctic	To evaluate the seasonal variation in daily step counts and sedentary time	Observational longitudinal prospective study (cohort)
		Average T (°C):		
		Summer (max): 27		
		Winter (min): −14		
Dėdelė et al. (2019) [32]	Lithuania (Kaunas)	Dfb: Warm summer humid continental − Average Temp. (°C) annual/summer/winter = 7.1–7.4/13.8/(−2) − Rain (mm) = 600–640/(≈335)/(≈305) − Sunlight (h) year ≈ 1870 − Snow (days) in winter = 65–80	To examine associations of seasonal PA levels with socioeconomic and health factors	Cross-sectional study
Furlanetto et al. (2017) [19]	Belgium (Leuven) and Brazil (Londrina)	Belgium: Cfb, Temperate oceanic	To quantify PA in daily life and compare its variability caused by seasonality	Observational longitudinal prospective study (cohort)
		Brazil: Cfa, Humid Subtropical		
		Average summer/winter (1) Belgium, (2) Brazil): − T (°C): (1) 19.1/2.8 (2) 24.3/16.1 − Rain (mm): (1) 1.3/3.4 (2) 6.3/3.3 − Sun light (min): (1) 903/571 (2) 760/651		
Hoaas et al. (2019) [15]	Norway (Tromso), Denmark (Esbjerg) and Australia (Melbourne)	Norway: Dfc, Subarctic	To examine differences in physical activity levels and establishing if any variations in physical activity were attributable to season	Cross-sectional study
		Denmark and Australia: Cfb, temperate oceanic		
		Temperature range (°C): − Norway: (−7)–15 − Denmark: (−2)–21 − Australia: 6–26		
Kim et al. (2016) [27]	USA (Southwest central region)	-	To examine the longitudinal trajectories in PA and its correlates	Observational longitudinal prospective study (cohort)
Kimura et al. (2015) [55]	Japan (Kahoku)	Cfa, humid subtropical	To compare the physical activity between summer and winter seasons	Observational longitudinal prospective study
		Average summer/winter: − Temperature (°C): 26.1/3.1 − Day length (h): 14.1/10.4 − Rain (mm): 7.83/5.08		
Klompstra et al. (2019) [45]	Sweden	Dfb/Dfc: Warm-summer humid continental/Subartic Temperature (°C) range: − Summer: 6/27 − Winter: −16/7	To describe the seasonal differences in physical activity	Cross-sectional study
Kong et al. (2020) [42]	South Korea (Seoul)	Dwa: Monsoon-influenced hot-summer humid continental	To examine how season and temperature levels affect physical activity-	Observational longitudinal prospective study (cohort)
		Average T (°C):		
		Summer (max): 30		
		Winter (min): −6		
Lapointe et al. (2016) [56]	Canada (Quebec)	ET/Dfc/Dfb: Tundra/Warm summer humid continental/Subartic	To evaluate seasonal variation on daily step counts	Observational longitudinal prospective study (cohort)
		Average T (°C):		
		Summer (max): 25		
		Winter (min): −15		
Nakashima et al. (2019) [13]	Japan (Gifu)	Cfa: Humid subtropical (Autumn/Winter/Spring/Summer): − Temperature (°C): 15.1/2.3/10.7/23.8 − Rain (mm): 11.2/7.7/2.5/6.2 − Sun Light (h): 4.0/3.8/5.5/5.0	To clarify the seasonal variation effects on PA	Cross-sectional study
Nioi et al. (2017) [57]	United Kingdom (Scotland)	Cfb: Temperate oceanic	To examine the variation of ligh exposure between season	Observational longitudinal prospective study (cohort)
		Average T (°C):		
		Summer (max): 18		
		Winter (min): −3		
Rockette-Wagner et al. (2016) [58]	-	-	To examine the effectiveness of a lifestyle intervention	Quasiexperimental pre-post study
Sayegh et al. (2016) [5]	Qatar	Bwh: Hot deserts	To assess the physical activity levels during 1-year	Observational longitudinal prospective study (cohort)
		Average T (°C):		
		Summer (max): 42		
		Winter (min): 13		
Shoemaker et al. (2016) [23]	USA	Dfb: Warm summer humid continental	To determine if seasonal variation in temperature affects daily PA	Randomized controlled trial
		Average T (°C):		
		Summer (max): 28		
		Winter (min): −11		
Shoemaker et al. (2019) [24]	USA (West Michigan)	Dfb: Warm summer humid continental	To determine the presence and magnitude of seasonal variation in daily PA	Retrospective longitudinal study
		Average T (°C):		
		Summer (max): 28		
		Winter (min): −11		
Urbański et al. (2020) [44]	Poland	Dfb: Warm summer humid continental	To assess the level of leisure-time physical activity (LTPA) and its differentiation across the seasons	Observational longitudinal prospective study (cohort)
		Average T (°C):		
		Summer (max): 24		
		Winter (min): −7		
Vaidya et al. (2018) [59]	France	Cfb: Temperate oceanic	To describe the evolution of physical activity parameters among COPD patients	Observational longitudinal prospective study (cohort)
		Average T (°C):		
		Summer (max): 27		
		Winter (min): 1		
Wan et al. (2017) [20]	USA (Boston)	Dfa: Hot summer humid continental	To examine the effect of season on daily PA (among other objectives)	Randomized controlled trial
		Average T (°C):		
		Summer (max): 28		
		Winter (min): −8		
Wesolowska et al. (2018) [17]	Poland	Dfb: Warm summer humid continental	To assess the level of activities of daily living and its differentiation by season	Cross-sectional study
		Average T (°C):		
		Summer (max): 24		
		Winter (min): −7		
Yu et al. (2018) [60]	Netherlands and Switzerland	Cfb: Temperate oceanic	To assess the impact of season on PROs/exacerbations of COPD	Observational longitudinal prospective study (cohort)
		Average T (°C): Netherlands/ Switzerland:		
		Summer (max): 22/24		
		Winter (min): 0/−4		

COPD = chronic obstructive pulmonary disease; GPAQ = Global Physical Activity Questionnaire; HF = heart failure; IPAQ-LF = International Physical Activity Questionnaire—Long Form; IPAQ-SF = International Physical Activity Questionnaire—Short Form; LAPAQ = LASA Physical Activity Questionnaire; LIPA= low-light PA; LSPA = lifestyle PA; LTPAQ-SCI = Leisure Time Physical Activity Questionnaire for persons with Spinal Cord Injury; MET = Metabolic Equivalent of Task; MVPA = moderate and vigorous PA; PA = physical activity; PASE = Physical Activity Scale for the Elderly; PRP= pulmonary rehabilitation program; SB = sedentary behavior; SBQ = Sedentary Behavior Questionnaire; SCI = spinal cord Injury; TEE = total energy expenditure.

**Table 2:** Population, variables and results of the included studies.

Author (Year)	Country	Population	Variables (Instrument)	Measurements Periods	Results
Adams et al. (2018) [53]	USA (Vermont)	*n* = 1476; 70.33% women; university students	Steps per day (smartwatch)	≥50 days.	Statistically significant variation: fewer steps in winter compared to spring and autumn.
Akande et al. (2019) [9]	Canada (Nunavut)	*n* = 272; 43.7% women; healthy adults Inuit and non-Inuit; age (mean ± SD) = 4.9 ± 12.6 years	Steps per day (pedometer)	1 week during summer months and 1 week during Winter months	Non statistically significant differences
Arnardottir et al. (2017) [37]	Iceland	*n* = 138; 61.1% women; older adults; age (mean ± SD) = 80.3 ± 4.9 years	Counts × min^−1^ (accelerometer) Considering SB, LIPA, LSPA, MVPA.	1 week during summer months and 1 week during winter months	Statistically significant differences more time during summer in all PA categories, except MVPA. SB was reduced in summer compared to winter
Carr et al. (2016) [16]	USA	*n* = 132; Spanish speaking women enrolled in a 12-month physical activity intervention age (mean ± SD) = 41.6 ± 10.1 years	− Steps per day − Moderate intensity aerobic steps (>100 steps/minute) (pedometer)	Every day for 12 months	Statistically significant differences were observed for both total steps and aerobic steps by season (summer > spring > fall > winter) in both groups
Cepeda et al. (2018) [34]	Netherlands (Rotterdam)	*n* = 1166; 56.6% women; three age groups: middle-aged (50–64 years), young-elderly (65–74 years) and old-elderly (≥75 years)	− Min/day Light PA − Min/day MVPA; − Min/day SB; (accelerometer)	7 days	Middle-aged and young-elderly → Statistically significant more light PA and MVPA in summer than winter No seasonal variations on SB. For old-elderly → non-significant seasonal variations
Collins et al. (2015) [31]	Canada (Ontario)	*n* = 1400 (350 per season); 64% women; age (mean) = 51 years	Active transportation (Phone questionnaire)	7 days before survey. Spring, autumn, summer and winter	Statistically significant lower in winter compared with other 3 seasons. Walking rates were highest in the fall and spring seasons, while cycling rates were highest in spring and summer
Cooke et al. (2016) [54]	Canada (Montreal)	*n* = 369; 54% women; adults with overweight/obesity and type II diabetes and/or hypertension; age (mean ± SD) = 59.6 ± 11.2 years	− Steps per day (accelerometer). − Proportion of sedentary time (accelerometer) − Sitting time: IPAQ-SF, SBQ	1 week spring/summer vs. autumn/winter	Statistically significant higher number of steps and less sedentary time in spring/summer compared to autumn/winter with objective methods. Non significative variations for subjective methods.
Dėdelė et al. (2019) [32]	Lithuania (Kaunas)	*n* = 1111; 57.7% women; age (mean ± SD) = 48.4 ± 16.8 years	− Self-reported commuting PA (walking and cycling) − Self-reported sufficient physical activity (>150 min per week) − Work and Leisure PA. (GPAQ)	Once in summer and once in winter	Statistically significant higher PA in summer tan winter.
Furlanetto et al. (2017) [19]	Belgium (Leuven) and Brazil (Londrina)	*n* = 37; Belgium (*n* = 18, 33% women, Brasil (*n* = 19, 47% women; COPD; age (mean) = 69 y	− Active time (time spent per day in activities of intensity >2 METs) − MVPA time: Time spent per day in MVPA (activities >3 METs) (Activity monitor)	7 days per season (summer and winter)	Active time: statistically significative decreased in winter compared to summer in both regions. MVPA: non statistically differences.
Hoaas et al. (2019) [15]	Norway (Tromso), Denmark (Esbjerg) and Australia (Melbourne)	*n* = 168; 42.8% women; patients with COPD moderate to severe; age (mean)= 66 years (Norway and Australia), 63 y (Denmark)	− Steps per day − TEE − Awake sedentary time − Light PA (1.5–3 METs), − Moderate-to-vigorous PA (≥3 METs). (Activity monitor)	7 consecutive days (1 cross-sectional observation/person)Seasons: winter, spring, summer or autumn	Non statistically significant differences among seasons. Clinically important difference of 600 steps/day: highest in summer compared with the other seasons
Kim et al. (2016) [27]	USA (Southwest central region)	*n* = 669; Women 3 age groups: 20–40 years (*n* = 83), 41–60 years (*n* = 394) y >60 years (*n* = 192)	Steps per day (pedometer)	18 consecutive months	Statistically significant increase in steps/day in spring. Significant decrease in autumn and winter. No significant change during summer periods.
Kimura et al. (2015) [55]	Japan (Kahoku)	*n* = 39; 56% women; Volunteer healthy older adults; Age (mean ± SD) = 70.7 ± 3.2 years	Step per day (pedometer)	7 days per season (two consecutive seasons for participant)	Statistically significant seasonal differences with higher average steps/day in summer than in winter
Klompstra et al. (2019) [45]	Sweden	*n* = 87; 29% women; Outpatients with HF;Age (mean ± SD) = 70 ± 9 years	METs per week (IPAQ-Short Form)	Once in summer and winter time	Non statistically significative differences PA
Kong et al. (2020) [42]	South Korea (Seoul)	*n* = 555; 43.7% women; preoperative lung cancer patients; age (mean ± SD) = 61.1 ± 8.9 years	− Steps per day − Min/day MVPA; (wearable activity tracker)	7 consecutive days (1 cross-sectional observation/person) in spring, summer, autumn or winter	Statistically significant seasonal differences on both variables: lower in winter compared to spring
Lapointe et al. (2016) [56]	Canada (Quebec)	*n* = 34; 44.1% women; participants with coronary heart disease; age (mean ± SD) = 67 ± 6 years; 2 groups by level of activity: Active (>7500 daily step), or Low active	Steps per day (pedometer)	1 week in each season: autumn, winter, spring, summer.	Active group: Statistically significant higher number of steps in spring and summer than in autumn and winter Low active: non-significant differences among seasons.
Nakashima et al. (2019) [13]	Japan (Gifu)	*n* = 22; 86% women in mountainous agricultural areas; Age (mean ± SD) = 75.1 ± 7.3 years	− Steps per day − TEE − Low and moderate- to high-intensity activities (accelerometer) − Daily activities performance (PASE Questionnaire)	1 year period with measurements in each season. Accelerometer: 7 day-period in each season	Statistically significant increase in steps/day (spring compared with winter) and in time spent in low intensity activities (higher in spring and summer than in winter). No seasonal variations on the PASE
Nioi et al. (2017) [57]	United Kingdom (Scotland)	*n* = 16; 81.2% women living in a care home; Age range = 72–99 years	− Active count/min − SB: active count/min < 100 (accelerometer)	4 days in 2 seasons: summer and winter	Statistically significant difference with higher PA in summer than in winter
Rockette-Wagner et al. (2016) [58]	-	*n* = 150; 91% women; adults with overweight/obesity;age (mean ± SD) = 51.1 ± 10.2 y	− Steps per day − Min/day Light PA − Min/day MVPA − Min/day SB (accelerometer)	winter, spring, summer, autumn	Statistically significant differences in number of steps, light PA, MVPA and SB: lower PA and higher SB in winter
Sayegh et al. (2016) [5]	Qatar	*n* = 549; adult women; age (mean ± SD) = 37.4 ± 11.7 years	− Daily total steps − Aerobic steps (pedometer)	One year period	Decrease in steps per day, in June, July, and August
Shoemaker et al. (2016) [23]	USA	*n* = 16; 56.2% men with heart failure and DCI/TRC devices; age (median) = 66 y	Daily minutes in activity level over 70 steps/minute (implanted accelerometer)	PA data available for 13–21 months	Statistically significantly higher PA in summer/autumn than in winter
Shoemaker et al. (2019) [24]	USA (West Michigan)	*n* = 168; 75% men; with heart failure and DCI/TRC devices; age (mean ± SD) = 63.0 ± 22.8 years	Daily minutes in activity level over 70 steps/minute (implanted accelerometer)	One year period. Bi-monthly data points (start of month and middle of month)	Statistically significative difference between the lowest PA in winter and highest in summer
Urbański et al. (2020) [44]	Poland	*n* = 51; 31% women; Participants with SCI; Age (mean ± SD) = 30 ± 7.9 years	Leisure-time physical activity (mild, moderate and heavy) (LTPAQ-SCI)	4 times/year (spring, summer, autumn and winter)	Statistically significant differences on mild and moderate LTPA (highest in spring, lowest in autumn) and heavy LTPA: (highest in summer, lowest in autumn)
Vaidya et al. (2018) [59]	France	*n* = 51; Patients with Age (mean ± SD) = 63 ± 9 years	Steps/day (actimeter)	1 week at the beginning and 1 week at the end of PRP	Statistically significant variation with higher amount of steps/day in summer compared to spring
Wan et al. (2017) [20]	USA (Boston)	*n* = 109; 98.5% men; U.S. Veterans with COPD in a RCT; Age (mean ± SD) = 68.6 ± 8.3 years	Steps/day (pedometer)	13 weeks. Seasons: spring, summer, autumn, winter.	Statistically significant decrease during the transition from summer to autumn, and significant increase in the transition from spring to summer.
Wesolowska et al. (2018) [17]	Poland	*n* = 106 volunteers; 59.4% women; three age groups: young (22–26 y), middle-aged (27–59 y), and senior (60–86 y)	− Steps/day (pedometer) − METs per week (IPAQ-LF)	Pedometer: 7 consecutive days per season IPAQ-LF: once per season	Statistically significant differences on steps/day: highest in summer and spring season in all study groups
Yu et al. (2018) [60]	Netherlands and Switzerland	*n* = 409; Patients with COPD	− PA Questionnaire (LASA PAQ)	Follow-up: 5 years Seasons: winter, spring, summer, autumn	Statistically significantly differences: higher level of PA in summer than winter

COPD = chronic obstructive pulmonary disease; GPAQ = Global Physical Activity Questionnaire; HF = heart failure; IPAQ-LF = International Physical Activity Questionnaire—Long Form; IPAQ-SF = International Physical Activity Questionnaire—Short Form; LAPAQ = LASA Physical Activity Questionnaire; LIPA= low-light PA; LSPA = lifestyle PA; LTPAQ-SCI = Leisure Time Physical Activity Questionnaire for persons with spinal cord injury; MET = metabolic equivalent of task; MVPA = moderate and vigorous PA; PA = physical activity; PASE = Physical Activity Scale for the Elderly; PRP= pulmonary rehabilitation program; SB = sedentary behavior; SBQ = Sedentary Behavior Questionnaire; SCI = spinal cord injury; TEE = total energy expenditure.

## Data Availability

Not applicable.

## Supplementary Materials

The following are available online at https://www.mdpi.com/article/10.3390/ijerph19010002/s1. Table S1: Prisma 2020 check list, Table S2: Systematic research, Table S3: Methodology quality assessment according to JIB checklist for cohort studies, Table S4: Methodology quality assessment according to JIB checklist for cross-sectional studies, Table S5: Methodology quality assessment according to JBI Checklist for Randomized Controlled Trials.
