# Supplementary material for: Impact of Seasonality on Physical Activity: A Systematic Review
Source: Int J Environ Res Public Health. 2021 Dec 21;19(1):2. doi: 10.3390/ijerph19010002 (PMC8751121; doi:10.3390/ijerph19010002)
Supplement: Supplementary file 1 [file ijerph-19-00002-s001.zip › Table S2. Systematic research.pdf]

Table S2. Systematic research.

MEDLINE	(((((motor activity[MeSH Terms]) OR physical activity[Title/Abstract]) OR exercise[MeSH Terms]) OR training[MeSH Terms]) AND seasons[MeSH Terms]) AND season*[Title/Abstract]
	physical activity[Title/Abstract]) AND season*[Title/Abstract]
PEDRO	Physical activity AND Season* (or Physical activity, Season*)
	Exercise AND Season* (or Exercise, Season*)
EMBASE (PICO search)	('motor activity'/exp OR 'physical activity':ti,ab,kw OR 'exercise'/exp OR exercise:ti,ab,kw) AND 'season'/exp AND season*:ti,ab,kw NOT sport*:ti,ab,kw NOT injur*:ti,ab,kw NOT athlete*:ti,ab,kw NOT hormon*:ti,ab,kw NOT vaccin*:ti,ab,kw NOT player*:ti,ab,kw NOT elite:ti,ab,kw NOT soccer:ti,ab,kw NOT 'vitamin d':ti,ab,kw NOT serum:ti,ab,kw NOT immune:ti,ab,kw NOT allergy:ti,ab,kw NOT influenza:ti,ab,kw NOT virus:ti,ab,kw AND [2015-2020]/py AND 'human'/de
COCHRANE	(Physical activity):ti,ab,kw AND (Season*):ti,ab,kw
