# Supplementary material for: Impact of Seasonality on Physical Activity: A Systematic Review
Source: Int J Environ Res Public Health. 2021 Dec 21;19(1):2. doi: 10.3390/ijerph19010002 (PMC8751121; doi:10.3390/ijerph19010002)
Supplement: Supplementary file 1 [file ijerph-19-00002-s001.zip › Table S3. Methodology quality assessment according to JIB cohort studies.pdf]

Table S3. Methodology quality assessment according to JIB checklist for cohort studies.

Author (year)	1	2	3	4	5	6	7	8	9	10	11	%
Akande et al. (2019)	Y	Y	Y	Y	U	Y	Y	Y	N	N	Y	72.7
Arnardottir et al. (2017)	Y	Y	Y	N	N	Y	Y	Y	N	N	Y	63.6
Furlanetto et al. (2017)	Y	Y	Y	N	N	Y	Y	Y	N	N	Y	63.6
Kim et al. (2016)	Y	Y	Y	N	N	Y	Y	Y	N	N	y	63.6
Kimura et al. (2015)	Y	Y	U	N	N	Y	U	U	N	N	Y	36.36
Kong et al. (2020)	Y	Y	Y	Y	Y	Y	Y	Y	N	N	Y	81.81
Nioi et al. (2017)	Y	Y	Y	U	N	Y	Y	Y	N	N	Y	63.6
Sayegh et al. (2016)	Y	Y	Y	N	N	Y	Y	Y	N	N	Y	63.6
Urbański et al. (2020)	Y	Y	Y	N	N	Y	Y	Y	N	N	Y	63.6

Y: YES, N: NO, U: UNCLEAR, %: PERCENTAGE
