# Supplementary material for: Impact of Seasonality on Physical Activity: A Systematic Review
Source: Int J Environ Res Public Health. 2021 Dec 21;19(1):2. doi: 10.3390/ijerph19010002 (PMC8751121; doi:10.3390/ijerph19010002)
Supplement: Supplementary file 1 [file ijerph-19-00002-s001.zip › Table S4. Methodology quality assessment according to JIB cross-sectional.pdf]

Table S4. Methodology quality assessment according to JIB checklist for cross-sectional studies.

**Author (year)**	**1**	**2**	**3**	**4**	**5**	**6**	**7**	**8**	**%**
Cepeda et al. (2018)	Y	Y	Y	Y	Y	Y	Y	Y	100
Collins et al. (2015)	Y	Y	Y	Y	N	N	Y	Y	75
Dėdelė et al (2019)	Y	Y	Y	Y	Y	Y	Y	Y	100
Hoaas et al. (2019)	Y	Y	Y	Y	Y	Y	Y	Y	100
Klompstra et al. (2019)	Y	Y	Y	Y	N	N	Y	Y	75
Nakashima et al. (2019)	Y	Y	Y	Y	N	N	Y	Y	75
Wesolowska et al. (2018)	N	N	Y	Y	N	N	Y	Y	50

Y: YES, N: NO, %: PERCENTAGE
