# Supplementary material for: Impact of Seasonality on Physical Activity: A Systematic Review
Source: Int J Environ Res Public Health. 2021 Dec 21;19(1):2. doi: 10.3390/ijerph19010002 (PMC8751121; doi:10.3390/ijerph19010002)
Supplement: Supplementary file 1 [file ijerph-19-00002-s001.zip › Table S5. Methodology quality assessment according to JIB checklist for randomized controlled trial.pdf]

Table S5. Methodology quality assessment according to JBI Checklist for Randomized Controlled Trials

**Author (year)**	**1**	**2**	**3**	**4**	**5**	**6**	**7**	**8**	**9**	**10**	**11**	**12**	**13**	**%**
Carr et al. (2016)	Y	Y	Y	N	N	Y	Y	Y	Y	Y	Y	Y	Y	84.62
Shoemaker et al. (2016)	Y	U	U	N	Y	Y	Y	U	U	Y	Y	Y	Y	61.54
Wan et al. (2017)	Y	Y	N	N	Y	Y	Y	U	Y	Y	Y	Y	Y	76.93

Y: YES, N: NO, U: UNCLEAR, %: PERCENTAGE
